# Revisiting the anatomy of the monocot cambium, a novel meristem

**DOI:** 10.1007/s00425-021-03654-9

**Published:** 2021-06-17

**Authors:** Joanna Jura-Morawiec, Alexei Oskolski, Philip Simpson

**Affiliations:** 1 Polish Academy of Sciences Botanical Garden - CBDC in Powsin, Prawdziwka 2, 02-973 Warsaw, Poland; 2 Department of Botany and Plant Biotechnology, University of Johannesburg, Auckland Park 2006, P.O. Box 524, Johannesburg, South Africa; 3 Botanical Museum, Komarov Botanical Institute of the Russian Academy of Sciences, Professor Popov str. 2, 197376 St. Petersburg, Russia; 4 Uruwhenua Botanicals, 51 Falconer Rd, Pohara RD 1, Takaka, New Zealand

**Keywords:** Initial cell, Secondary growth, Semi-storied cambium, Tree-like monocots, Vascular cambium

## Abstract

**Main conclusion:**

The monocot cambium is semi-storied, and its cells do not undergo rearrangement.

**Abstract:**

The monocot cambium is a lateral meristem responsible for secondary growth in some monocotyledons of Asparagales. It is an unusual meristem, not homologous with the vascular cambia of gymnosperms and non-monocotyledonous angiosperms. Owing to the limited information available on the characteristics of this meristem, the aim of this study was to survey the structure of the monocot cambium in order to clarify the similarities and dissimilarities of this lateral meristem to the vascular cambium of trees. Using the serial sectioning analysis, we have studied the monocot cambium of three species of arborescent monocotyledons, i.e., Quiver Tree *Aloe dichotoma*, Dragon Tree *Dracaena draco*, and Joshua Tree *Yucca brevifolia*, native to different parts of the world. Data showed that in contrast to the vascular cambium, the monocot cambium is composed of a single type of short initials that vary in shape, and in tangential view display a semi-storied pattern. Furthermore, the cells of the monocot cambium do not undergo rearrangement. The criteria used in identifying monocot cambium initial cell are also discussed.

## Introduction

During the course of evolution, the two secondary vascular meristems have developed for the radial growth of plant organs, namely the vascular cambium and the monocot cambium (Spicer and Groover 2010; Carlquist 2012). Both meristems are concentric and contain cambial initials that undergo periclinal divisions, and these, in turn, differentiate to produce secondary vascular tissues (Evert 2006; Jura-Morawiec et al. 2015). The vascular cambium, characteristic of gymnosperms and non-monocotyledonous angiosperms, arises between the primary xylem and phloem, mainly from procambium, and contains fusiform and ray initial cells that form secondary phloem and secondary xylem, i.e., wood (Larson 1994). Conversely, the monocot cambium which occurs in some monocotyledons of the order Asparagales, such as *Aloe*, *Dracaena* and *Yucca* (Rudall 1995), is formed outside the primary vascular bundles from the primary thickening meristem (Diggle and DeMason 1983a, b; Stevenson and Fisher 1980; Rudall 1991) or pericycle (Cattai and Menezes 2010), and contains a single type of initial cells (Cheadle 1937; Philipson et al. 1971). Its derivatives form the secondary cortex centrifugally, and centripetally secondary ground tissue with secondary xylem and phloem arranged in collateral or amphivasal vascular bundles (Tomlinson and Zimmermann 1967). Thus, the vascular cambium and monocot cambium have similar roles in radial growth but differ in establishment of the cambial cylinder, cell composition and the nature of their derivatives.

The monocot cambium is not considered to be homologous with the vascular cambium (Rudall 1995), and has been termed a ‘novel meristem’ (Spicer and Groover 2010; Zinkgraf et al. 2017). Nearly a hundred years ago, Cheadle (1937) pointed out the lack of information available in the literature concerning monocot cambium initials. However, only Simpson (1975) subsequently took up this challenge, and based on studies of the monocot cambium of *Yucca brevifolia*, proposed that cell shape, lack of intercellular spaces and the thicker tangential wall of the terminal cell of a radial file may prove helpful in identifying these meristematic cells. In general, however, the monocot cambium structure has received relatively little attention. Descriptions of secondary growth in monocots focus mainly on derivative tissues (Jura-Morawiec et al. 2015; Maděra et al. 2020) hampering our understanding of monocot cambium organization. On the other hand, the recent findings of Zinkgraf et al. (2017) have shown a considerable overlap in gene expression between the monocot cambium and the vascular cambium. Thus, the concept of monocot cambium remains obscure.

The aim of this study is to survey the structure of the monocot cambium of the tree-like representatives of Asparagales in order to clarify the similarities and dissimilarities of this lateral meristem to the vascular cambium of gymnosperm and non-monocotyledonous angiosperm tree species. We compared the size, shape, and arrangement of the cambial cells of three monocot species native to different parts of the world. The criteria used to identify monocot cambium initials are also discussed on this basis.

## Materials and methods

### Plant material

**Fig. 1:**
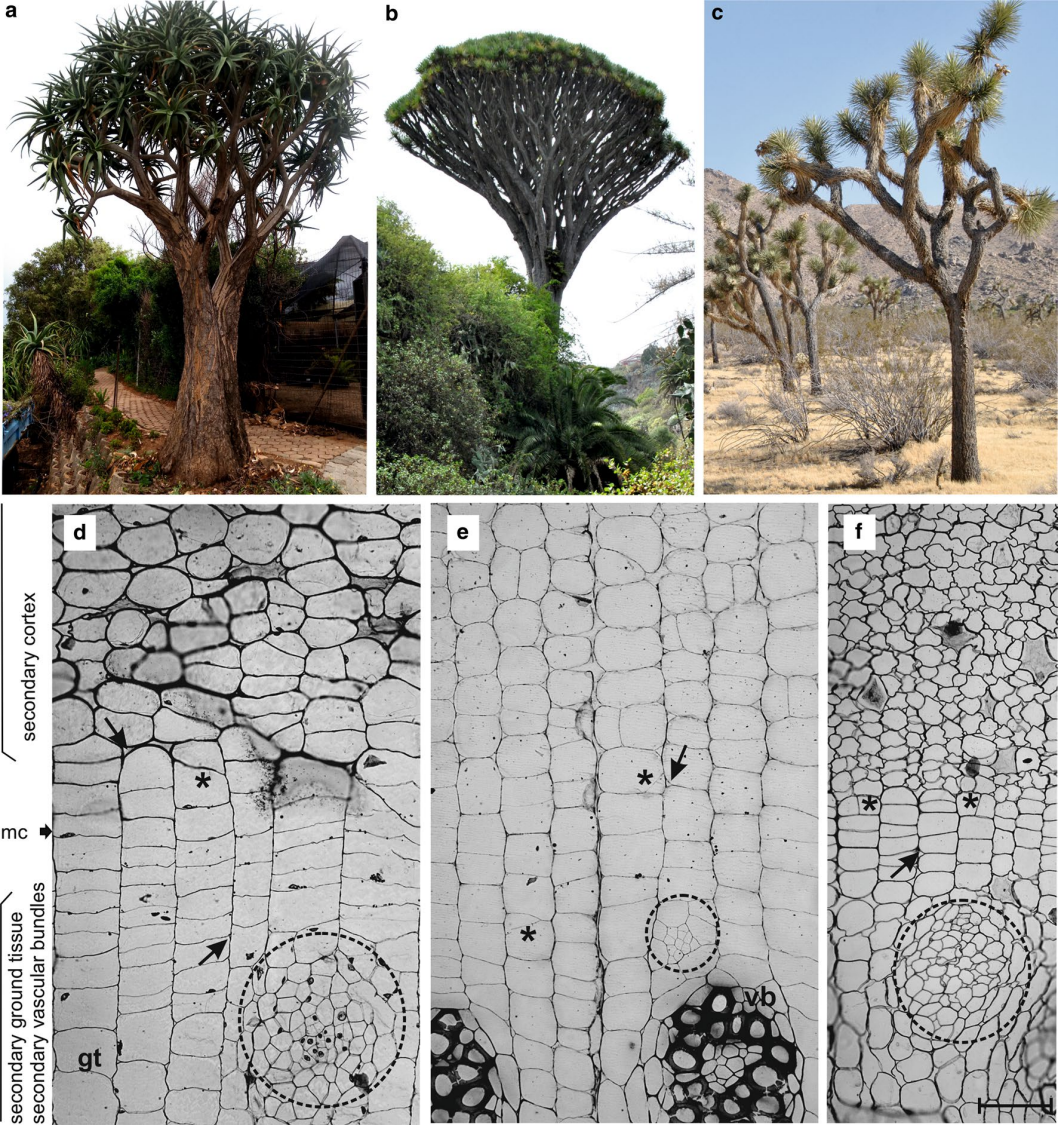
Examples of arborescent monocotyledons. **a** *Aloe dichotoma*, South Africa. **b** *Dracaena draco*, Gran Canaria, Spain. **c** *Yucca brevifolia*, Mojave Desert, USA. **d–f** Some details of the respective secondary growth. A thick arrow indicates possible location of the monocot cambium (mc); thin arrows and asterisks indicate intercellular spaces and anticlinal divisions outside the cambium, respectively. gt, secondary ground tissue; vb, secondary vascular bundle; developing secondary vascular bundles are encircled. Scale bar = 100 µm, valid for sections **d–f**

For the purpose of the study, samples of the monocot cambium were taken from three species of arborescent monocots (Fig. 1a–c). The first was a Quiver Tree (*Aloe dichotoma* syn. *Aloidendron dichotomum*, Asphodelaceae), native to Northern Cape and Namibia (van Jaarsveld 2011; Cousins and Witkowski 2012; Guo et al. 2016). The samples were taken from an individual growing in Walter Sisulu National Botanical Garden located in Johannesburg, South Africa. The stem girth at the sampling point was 167 cm. The second monocot was a Dragon Tree (*Dracaena draco,* Asparagaceae), native to the Canary Islands, Madeira and Morocco (Marrero et al. 1998; Maděra et al. 2020). Samples were taken from three individuals growing in the natural vegetation belt for this species, a thermosclerophyllous forest at the Botanical Garden “Viera y Clavijo” on Gran Canaria (Spain). Their girth at the point of sampling was 111, 113 and 125, respectively. The third arborescent monocot was a Joshua Tree (*Yucca brevifolia* var. *brevifolia,* Asparagaceae), native to the Mojave Desert of southwestern USA (Gilliland et al. 2006). Samples were taken from two individuals (circa 98 and 101 cm in girth) growing in Yucca Valley, located in southern California’s San Bernardino County.

### Microscopical analysis

Samples were fixed in FAA or glycerol:ethanol (1:1) and stored in 70% (v/v) ethanol. After cutting these into smaller pieces, they were embedded in epon resin (Sigma-Aldrich) according to the standard protocol (Jura-Morawiec 2017). Next, transverse, tangential and radial sections were cut at a thickness of 3.5 µm using a Leica 480A microtome, attached to glass microscope slides with Haupt adhesive and stained with PAS (Periodic Acid, Schiff) and toluidine blue, and mounted in Euparal (Roth). The preparations were then examined using an Olympus BX41 light microscope equipped with a Canon EOS 70D camera.

To study cell shape, cell arrangement and the occurrence of cell events (intrusive growth, symplastic growth, and cell divisions), numerous transverse and longitudinal sections were used, together with serial sectioning analysis. Cambial cell dimensions (length and width) were measured based on micrographs of tangential sections. Owing to the fact that cambial cells are polygonal in shape, the left–right and basal–apical axes were used to measure cell width and length, respectively (see Fig. 3b). One hundred cambial cells randomly selected for each species were measured with ImageJ. The term monocot cambium will be used here in a broad sense to include the initials and its recent derivatives. As long as these cells remain undifferentiated (i.e., before any evidence of anticlinal divisions, lack of intercellular spaces, lack of enlargement and lack of formation of the secondary wall), the cells should remain fairly representative of the cambial cells. The width of cambium is reported here in terms of cell number in a radial file. Cambial cells occur in horizontal tiers in tangential view when the ends of cells of one tier appear at approximately the same level. Intrusive growth is considered here as growth of cell tips which involves the formation of new contacts between cells and leads to a change in the existing position of cells (i.e., leads to rearrangement, e.g., Jura et al. 2006). Secondary cortex was distinguished based on the combination of the following criteria (i) abundant calcium oxalate crystals, (ii) cells arranged in radial files, (iii) no vascular bundles (apart from horizontal leaf traces, which are continuous to the outer surface of the protective tissue), in marked contrast to the primary cortex where many bundles terminate or fuse to others (Simpson 1975).

## Results

### General characteristics of the monocot cambium

**Fig. 2:**
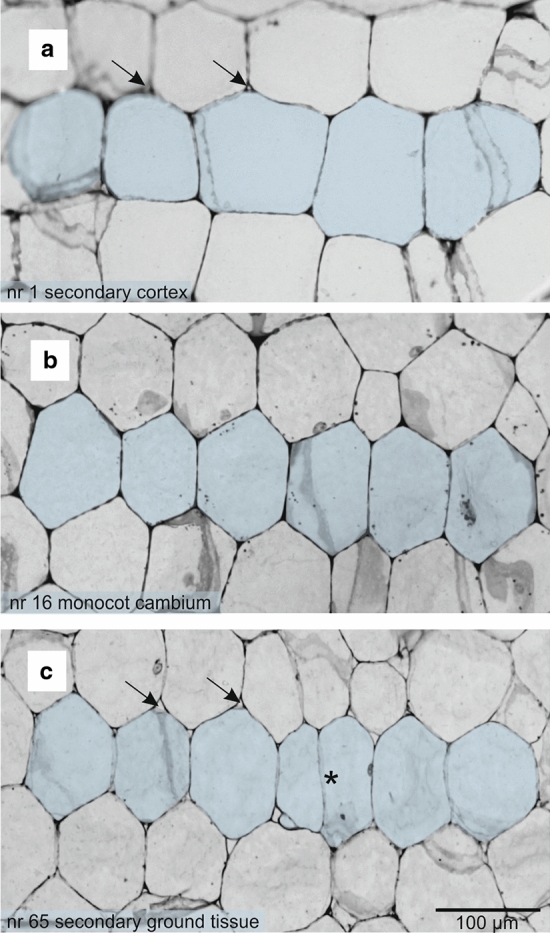
The monocot cambium and its derivative tissues in tangential view based on an *A. dichotoma* stem. **a–c** Selected successive tangential sections from a series of 65 sections covering a radial distance of 227.5 µm that included a tier (blue) of secondary cortex cells (**a**), cambial cells (**b**), and secondary ground tissue cells (**c**). In the lower left corner is the section number for the series. Note the slight changes in cell length and cell shape. Arrows indicate intercellular spaces; an asterisk shows anticlinal division in early vascular bundle differentiation

In transverse sections of the stem, the monocot cambium consists of flattened cells organized into radial files (Fig. 1d–f). Cambial cells divide by periclinal divisions. Anticlinal divisions were not observed in investigated monocot cambium cells. The cambium is narrow (2–4 cells) in *Y. brevifolia*, and wider in the two other species, up to 8 cells in *D. draco* (Table 1; Fig. 1d–f). The outer edge of the cambium, adjacent to the secondary cortex, is clearly demarcated in *Y. brevifolia* and *A. dichotoma*, where cells of the cortex are isodiametric, or of irregular shapes when desiccated, and not aligned in radial files (Fig. 1d–f), in contrast to the cambium of *D. draco* (Fig. 1e). The transition with the central cylinder is more complex and less defined in all investigated species. There, some of the cells divide in all vertical planes and produce vascular bundle, while other cells usually enlarge radially (becoming secondary ground tissue cells), and usually clear intercellular spaces between them are visible (Figs. 1d–f, 2c).

**Table 1:** Quantitative parameters of the monocot cambium

Trait	*Aloe dichotoma*	*Dracaena draco*	*Yucca brevifolia*
Cambial cell length (µm)^a^	109.44 (79–138)	119.57 (80–165)	72.52 (53–89)
Cambial cell width (µm)^a^	81.59 (44–126)	73.48 (46–94)	53.7 (33–72)
Width of cambium (number of cells)^b^	2–7	3–8	2–4
Number of cells in stories^b^	2–10	2–7	2–9

^a^*n* = 100

^b^*n* = 20

### Shape and size of cambial cells

**Fig. 3:**
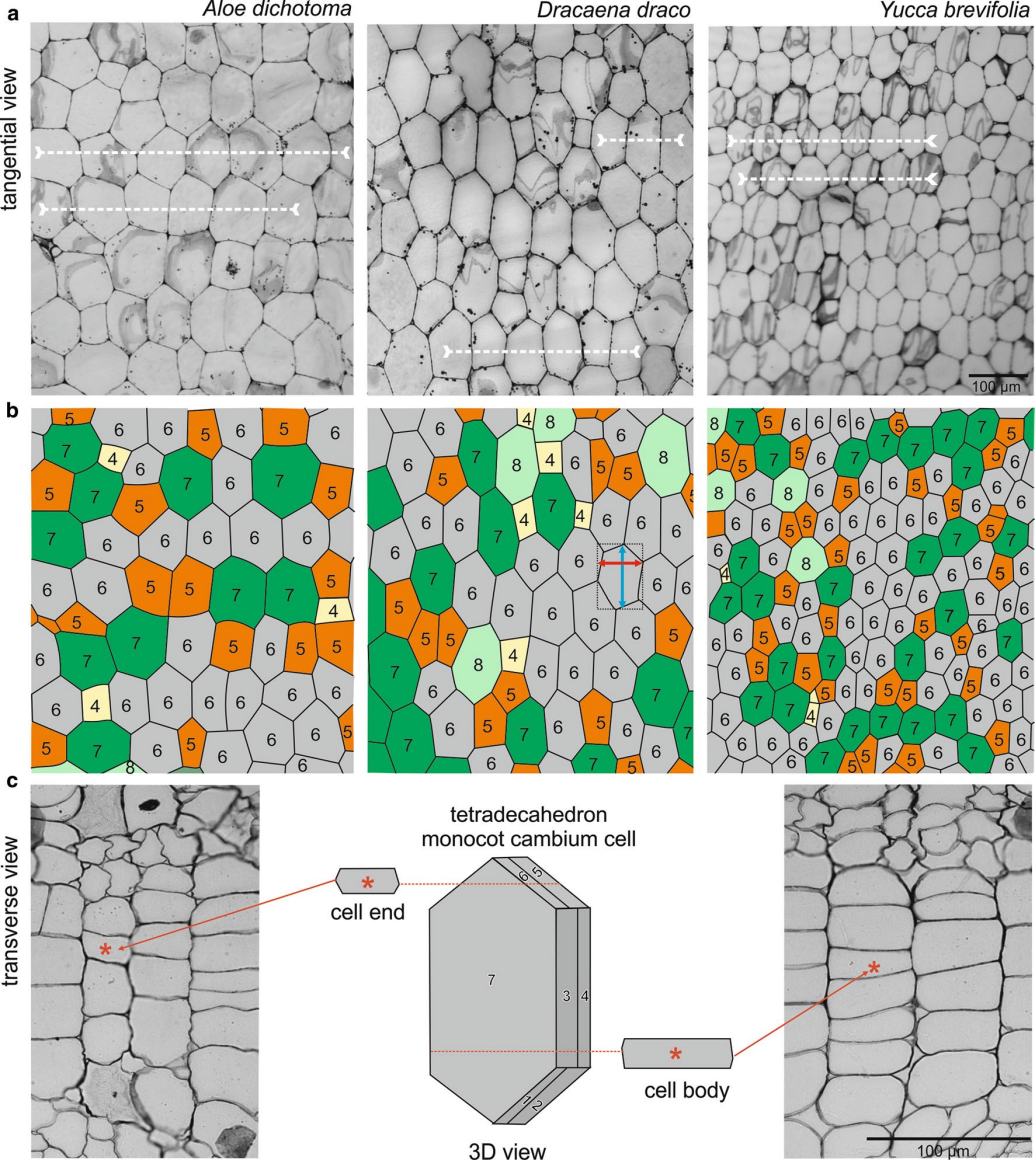
Characteristics of the shape and arrangement of cambial cells of three arborescent monocots. **a** Tangential sections through the monocot cambium of *A. dichotoma*, *D. draco*, and *Y. brevifolia* showing the semi-storied arrangement. Examples of stories are indicated with dotted horizontal lines. **b** Drawings made on the basis of sections with polygonal (2D) representation of cells. Monocot cambium cells are represented as *n*-sided polygons depending on the number of sides they possess, i.e., 4-sided (yellow), 5-sided (orange), 6-sided (gray), 7-sided (green), 8-sided (light green). In the central scheme, the way of measurement of the polygonal cell (length and width) is illustrated. **c** Scheme showing three-dimensional shape of the monocot cambium cell (14-sided; seven contacts marked 1–7 are visible, but the remaining seven are not), and corresponding views of the ends and body regions of cells as viewed in transverse sections through the monocot cambium of *Y. brevifolia*

Cambial cells have two characteristic features that help distinguish them from the derivatives. Serial sectioning analysis along the cortex (Fig. 2a), through the cambial cells (Fig. 2b), to the secondary ground tissue (Fig. 2c) reveals that cells of the monocot cambium have pointed ends and no or very reduced intercellular spaces at their corners. By contrast, cells of the secondary cortex and central cylinder, although similarly variable in appearance, have rounded corners with conspicuous intercellular spaces. Monocot cambium cells vary from rectangular to polygonal in shape. In tangential section (two dimensional), each cambial cell can be considered as an *n*-sided polygon, where *n* determines the number of neighbors it possesses (Fig. 3a, b). Thus, cambial cells may be 4-, 5-, 6-, 7- or 8-sided polygons. As seen in transverse view cells overlap by one-third in alternate radial files (Figs. 1d–f, 3c). As a result, in three-dimensions a cambial cell may possess 10 (a decahedron) to 18 (an octadecahedron) faces, the most frequent being 14 (a tetradecahedron) (Fig. 3c). These cells are relatively short (Table 1), but rather taller than broad.

### Arrangement and rearrangement of cambial cells

The organization of cambial cells in tangential view shows a semi-storied pattern. These cells thus form a mosaic where they occur locally in few-celled horizontal tiers (Figs. 2b, 3a; Table 1). Most of the cells contributing to these are 6-sided (hexagonal), as seen in the section, and are thus tetradecahedra when considered in three dimensions (Fig. 3c).

No evidence of intrusive growth in the cambium was observed. Thus, the cambial cells do not undergo rearrangement. In transverse section, differences in the tangential width of radial files are due to the height at which the cambial cells are cut, i.e., whether the microtome knife passes through the body or through the ends of the cell (Fig. 3c). However, considering the variable shape of cambial cells and their general lack of uniformity when viewed in tangential section (Fig. 3a), narrow files may also be due to the knife passing through relatively smaller and thinner 4-sided cells (hexagons as seen in section, decahedra when considered in three dimensions).

## Discussion

### Monocot cambium *versus* vascular cambium

The vascular cambium and monocot cambium have similar roles but differ in how they are formed, cell composition and the nature of their derivatives (e.g.,Cheadle 1937; Tomlinson and Zimmermann 1967; Diggle and DeMason 1983a, b; Stevenson and Fisher 1980; Rudall 1991; Cattai and Menezes 2010; Jura-Morawiec et al. 2015). Our results, combined with literature studies, indicate some other differences (Table 2). In terms of cell shape, cell arrangement and size, the differences are substantial. Whereas the vascular cambium has two types of cambial initials, the fusiform and ray initials, which in tangential view appear to be arranged in non-storied, semi-storied, storied or double-storied patterns (Larson 1994; Kojs et al. 2004a, b), the monocot cambium has only one type of initials (Cheadle 1937). As our results show, these initials may be arranged in a semi-storied pattern. This pattern probably results from anticlinal divisions that occurred during the formation of the monocot cambium from the primary thickening meristem, where anticlinal divisions account for the rapid expansion of the stem (Simpson 1975). Some monocot cambial cells are rectangular, whereas others are polygonal and truncated at one end, but pointed at the other (Cheadle 1937; Philipson et al. 1971). Taking into account that cambial cells overlap by one-third in alternate files their shape in three-dimensions may vary from decahedra to octadecahedra.

**Table 2:** Comparative characteristics of the monocot cambium and vascular cambium

Criterion	Monocot cambium	Vascular cambium
Establishment of cambial cylinder	between the primary cortex and the primary vascular bundles^a,b,c,d^	between the primary phloem and xylem^e^
Types of initials	single type of initials^a^	two types (fusiform initials and ray initials)^e^
Production of vascular derivatives	unidirectional; secondary phloem and secondary xylem deposited centripetally to the cambial cylinder and organized in secondary vascular bundles embedded in a ground tissue^a,b,c,d^	bidirectional; secondary phloem deposited centrifugally, and secondary xylem deposited centripetally to the cambial cylinder, vascular tissues are spatially separated^e^
Function	*secondary growth*^a,b,c,d^	*secondary growth*^e^
Cell shape in tangential section	from rectangular through *hexagonal* to polygonal^a,b,d^	*hexagonal* fusiform initials and isodiametric ray initials^e^
Cell shape in transverse section	*hexagonal*^a,b,c,d^	*hexagonal*^e^
Cell shape in radial direction	*hexagonal*^a,b,c,d^	*hexagonal* fusiform initials and procumbent or upright ray initials^e^
3D shape of cells	from decahedral through *tetradecahedral* to octadecahedron	*tetradecahedral* fusiform initials, cuboid ray initials^e^
Primary pit-fields	*present*^b^	*present*^e^
Cells arrangement	*semi-storied*	nonstoried, *semi-storied*, storied, double-storied^e^
Length of cells	40–165 µm	~ 170–8700 µm^e,f^
Cell events	*periclinal divisions*^b^, *anticlinal divisions*^b^ *symplastic growth*	*periclinal divisions*^e,f,g,h,i^, *anticlinal divisions*^e,f,g,h,i^, *symplastic growth*^e,g,h,i^, intrusive growth/elimination^e,g,h,i^
Cells rearrangement	absent	present^e,g,h,i^
Increase in cambium width	*symplastic growth of cambial cells in radial direction*	*symplastic growth of cambial cells in radial direction*^j^
Increase in cambium circumference	*symplastic growth of cambial cells in circumferential direction*	*symplastic growth of cambial cells in circumferential direction*^j^

Similarities are in italics

^a^Cheadle (1937)

^b^Simpson (1975)

^c^Rudall (1991)

^d^Jura-Morawiec et al. (2015)

^e^Larson (1994)

^f^Bailey (1923)

^g^Kojs et al. (2004b)

^h^Jura et al. (2006)

^i^Włoch et al. (2013)

^j^Miodek et al. (2021)

In general, cambial cell length is a species-specific character (Larson 1994). For *Agave americana, Aloe saponaria, Dasylirion serratifolium* and *Yucca glauca*, they vary from 50 to 75 µm (Cheadle 1937). These values are considerably smaller than ours for *D. draco* (80–165 µm) and *A. dichotoma* (79–138 µm)*.* Nevertheless, comparing our results for cambial cell length in *Y. brevifolia* (53–89 µm) reveals that they are rather similar to those (40–60 µm) reported by Simpson (1975). Admittedly, our measurements were taken for only a small number of individuals, since plant material is difficult to obtain, owing to the endemic status of the species involved. However, according to Simpson (1975), the average size of cambial cells shows no correlation with stem diameter but differs from stem to stem.

The length of initials reflects the type of anticlinal division involved. As a general rule, species with short fusiform initials tend to divide by means of radial anticlinal walls, whereas those with long initials divide obliquely (Larson 1994). Radial and oblique anticlinal divisions are relatively rare in the monocot cambium (Simpson 1975). Transverse divisions are absent in this meristem (Simpson 1975), but do occur in its centripetal derivatives which differentiate to form vascular bundles. Radial anticlinal divisions may occur in cambial cells as a prerequisite for symplastic growth with its associated increase in cambial circumference. This is defined as a uniform, coordinated growth process that does not involve a change in contacts between adjacent cells (Erickson 1986). Thus, the circumferential symplastic growth of cambial cells is contingent on the increase in the radius of the cambial ring (Karczewska et al. 2009; Miodek et al. 2021). Since the rate of growth in stem thickness of a woody plant diminishes with age (Bannan 1962), anticlinal divisions in old trunks are extremely rare, and this agrees with our observations. For example, trunk diameter of a 50–100 year old *D. draco* increases at a rate of about 1.0 cm per year, whereas the diameter of a young stem increases at a rate of 4.0 cm per year (Symon 1974).

Another difference between the vascular cambium and the monocot cambium is that of cell rearrangement. The initial cells of the vascular cambium grow intrusively, changing cell contacts and affecting the formation of wood grain (Harris 1989; Larson 1994; Lev-Yadun 2001; Włoch et al. 2002; Kojs et al. 2004a, b). In contrast, the initials of the mature monocot cambium do not grow intrusively and do not rearrange. Therefore, the monocot “wood”, apart from having a completely different structure, shows also no association between the direction of alignment of cells relative to the longitudinal axis of the stem. Intrusive growth is restricted only to the cambial derivatives i.e., developing tracheids within the vascular bundles which may be several orders of magnitude (forty times or more) longer than the cambial cells (Scott and Brebner 1889; Jura-Morawiec 2017).

**Fig. 4:**
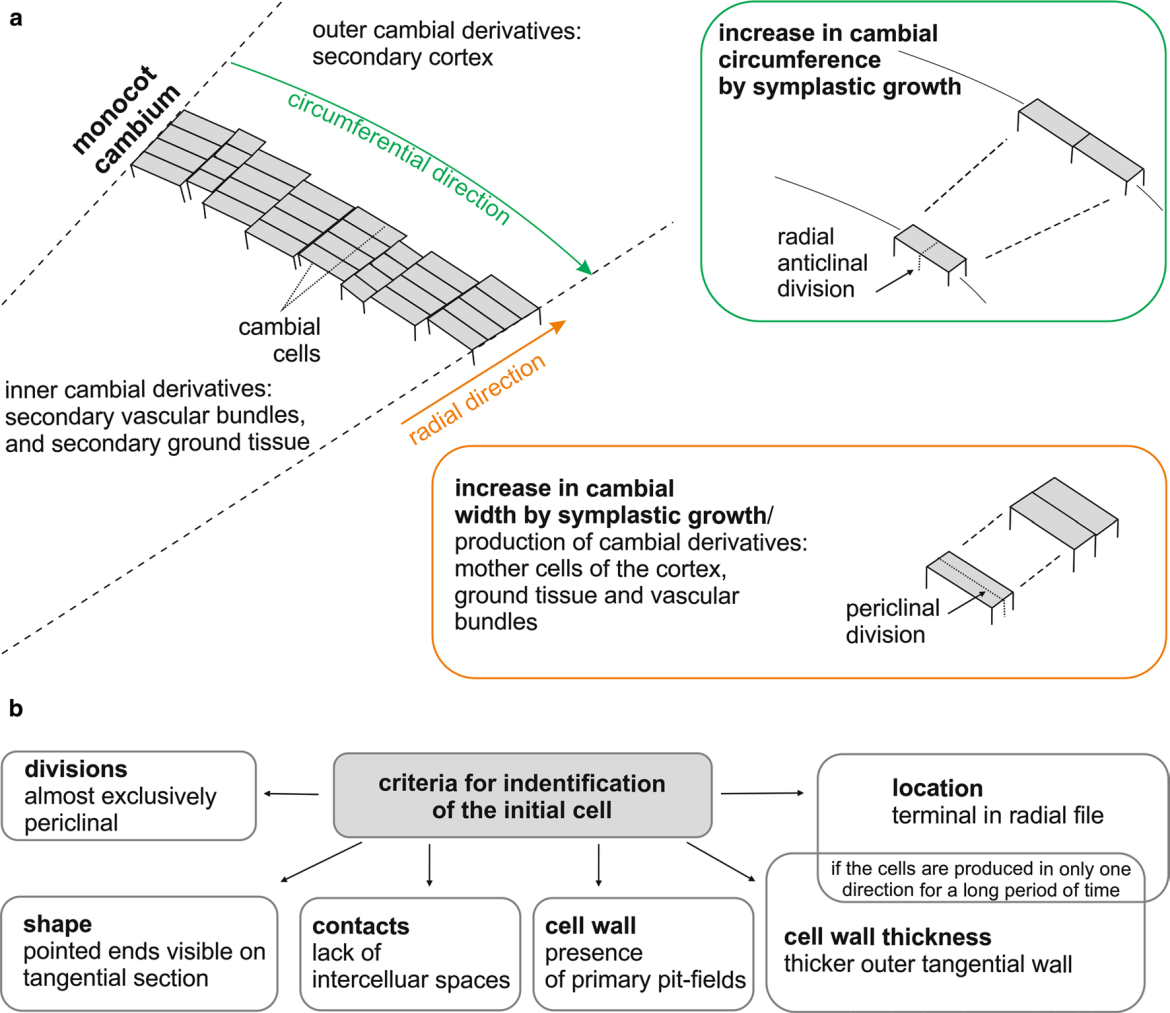
The organization of the monocot cambium. **a** An increase in monocot cambium circumference is the result of symplastic growth in circumferential direction (green box) whereas an increase in the width of the monocot cambium is the result of symplastic growth in radial direction (orange box). **b** Criteria for possible identification of the initial cell of the monocot cambium based on the work of Simpson (1975)

Despite some remarkable differences between the structure of the monocot cambium and that of the vascular cambium (cellular composition, and lack of cell rearrangement), the general design and function of the meristems are similar. In transverse section, the meristem is cylindrical in shape and its initials and recent derivatives are arranged in radial files (Fig. 4a). The main direction of growth of cambium cells is radial, and the main type of division is periclinal. The width of the cambium varies according to the rate of differentiation and number of cell divisions (Larson 1994; Jura-Morawiec 2015). Cambial circumference is modified by symplastic growth (Fig. 4a).

### Location of initial cell

The vascular cambium contains initial cells that maintain the integrity of the meristem, but are anatomically indistinguishable from their derivatives (Larson 1994; Evert 2006). The concept of the initial cell is based on its development, not its structure. Unlike its derivatives which will eventually leave the meristem and differentiate into permanent tissues, the initial cells will remain within the cambium. Taking into account the analysis of cell events (periclinal divisions, intrusive growth), the cambial initial is located in the radial file in a four-celled complex named Sanio’s four (Larson 1994), situated closer to the phloem during the period of greatest vascular cambium activity (Bannan 1962), and growing intrusively during cambial cell rearrangement that is recorded in wood structure (Włoch et al. 2002; Kojs et al. 2004a, b; Jura et al. 2006; Wilczek et al. 2011). Cambial initials may also develop thick walls with deeply depressed primary pit-fields (Esau 1965).

**Fig. 5:**
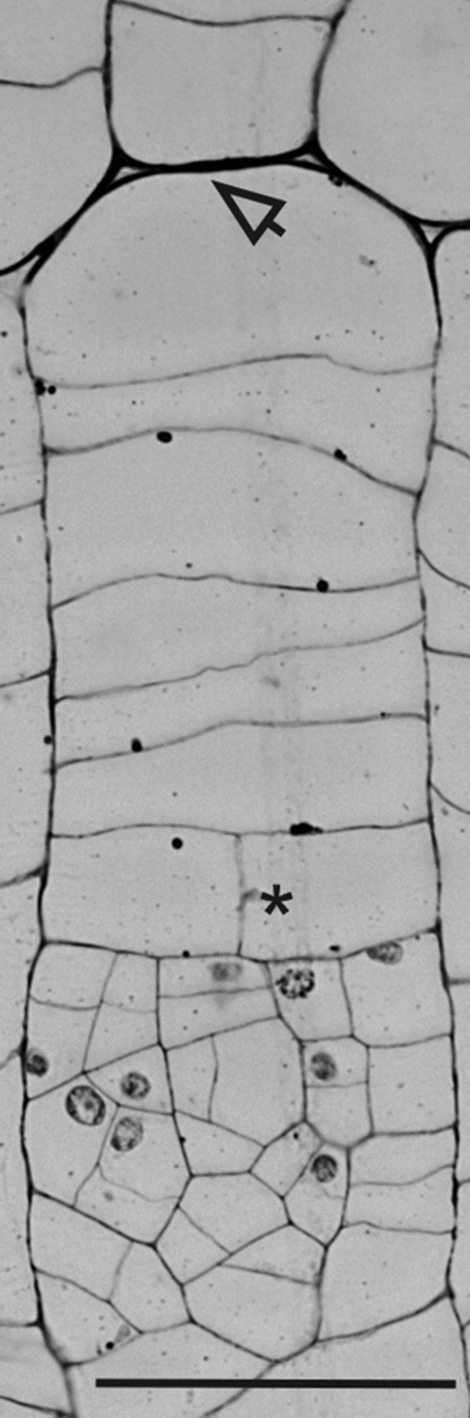
*A. dichotoma,* transverse section of a radial file of monocot cambium. The arrow indicates a thicker, tangential wall adjacent to the secondary cortex. The asterisk indicates an anticlinal cell division, and below this is an early stage in secondary vascular bundle development. Scale bar = 100 µm

The initial cell of the monocot cambium is also anatomically indistinguishable within a radial file. However, according to Simpson’s (1975) report for *Y. brevifolia*, a number of criteria, when used in combination, can help identify it or at least do so to a group of 2–3 cells (Fig. 4b). The initial cell of the monocot cambium divides almost exclusively by periclinal divisions. If the cambial cell produces derivatives alternately first centripetally and then centrifugally, it will possess thin walls. However, if the initial produces cells either centripetally or centrifugally for a prolonged period, it produces an increasingly thicker wall in that area (Fig. 5). Relatively thick periclinal walls may occur in the cambial cells, and it is probable that the initial cell is located next to this wall and is usually the terminal cell of a radial file. This is not a precise criterion since sooner or later this thicker wall will appear at the side associated with the secondary cortex or secondary ground tissue. Another criterion involves cell shape and its contacts. Only cambial cells have pointed ends visible in tangential section and thus lack or have very reduced intercellular spaces at their corners. Monocot cambium cells also have primary pit-fields in their walls. Our studies confirmed the universal nature of these criteria, however, even when used in combination, although they allow the general identification of monocot cambial cells, they do not enable the precise identification of the initial cell.

To conclude, some monocotyledonous plants have uniquely achieved the ability to grow in girth by means of a remarkable secondary vascular meristem referred to as the monocot cambium. Although its function is the same as that of the vascular cambium of gymnosperms and non-monocotyledonous angiosperms, the monocot cambium differs in establishment, its composition, and its derivative tissues. Like the vascular cambium, in transverse section, the monocot cambium is seen to form a ring that increases in both width and circumference owing to symplastic growth of the cambial cells following periclinal and anticlinal divisions, respectively. The initial cell of the monocot cambium is also anatomically indistinguishable. However, unlike the vascular cambium, cells of the monocot cambium are shorter, vary in shape, and in the mature monocot cambium may occur only in a semi-storied pattern. Finally, cells of the monocot cambium do not grow intrusively, and therefore, do not undergo rearrangement.

## Data Availability

All data generated or analyzed during this study are included in this published article.
